# World TB Day — March 24, 2020

**DOI:** 10.15585/mmwr.mm6911a1

**Published:** 2020-03-20

World TB Day is observed each year on March 24, providing an opportunity to increase awareness about tuberculosis (TB) and the actions needed to find, treat, and prevent this devastating disease.

In 2019, a provisional total of 8,920 TB cases were reported in the United States (incidence = 2.7 cases per 100,000 persons) (*1*), a 1.1% decrease from the 9,021 cases reported during 2018 and the lowest number of U.S. cases recorded since reporting began in 1953. Increased diagnosis and treatment of latent TB infec­tion remains essential for eliminating TB in the United States.

An analysis of global TB surveillance data found that in 2018, an estimated 10 million persons with incident TB and 1.5 million TB-related deaths occurred worldwide, represent­ing 2% and 5% declines from 2017. Among the estimated 10 million persons with TB, 70% were reported to WHO in 2018, a 9.4% increase from 2017 (*2*). Approximately 862,000 reported TB cases occurred among persons living with human immunodeficiency virus (HIV) infection. In 2018, 1.8 million persons with HIV began TB preventive treatment (TPT), a 88% increase in treatment initiation from 2017. Less progress in TPT implementation was reported among children aged <5 years than among persons living with HIV infection. TPT has been demonstrated to decrease morbidity and mortality among persons with HIV infection. Full implementation of effective strategies, including TPT, is crucial for reaching global targets.

CDC is working with partners to diagnose, treat, and prevent TB in the United States and globally. Additional information is available at https://www.cdc.gov/tb/worldtbday/ and https://www.cdc.gov/globalhivtb/who-we-are/events/world-tb-day/worldtbday.html.
